# Supplementary material for: Telomere Length and Mitochondrial DNA Copy Number Variations in Patients with Obesity: Effect of Diet-Induced Weight Loss—A Pilot Study
Source: Nutrients. 2022 Oct 14;14(20):4293. doi: 10.3390/nu14204293 (PMC9610454; doi:10.3390/nu14204293)
Supplement: Supplementary file 1 [file nutrients-14-04293-s001.zip › nutrients-1967249-supplementary.pdf]

Supplementary Table S1. Higher-TL (H-TL, N=9) and Lower-TL (L-TL, N=8) patient groups at baseline (T0).

	**H-TL (T0)**	**L-TL (T0)**	**p-value**
Age (years)	48±11.5	53.5±11.5	0.32
Weight (Kg)	98.3±13.4	101.3±13	0.64
Heigt (cm)	158.5±9.1	159.2±9.1	-
Waist (cm)	116.2±9.4	119.5±8.2	0.46
BMI (Kg/m^2)	39.1±3.5	39.9±3.9	0.66
Fat Mass (%)	46.3±5.9	44.6±6.5	0.57
Fat Free Mass (%)	53.7±5.9	55.4±6.5	0.58
Fasting Glucose (mg/dL)	85.9±10.7	92.1±7.0	0.43
HbA1c (mmol/mol)	37.4±4.5	40.3±7.6	0.36
HOMA (mmol/L × µU/m)	2.6±1.2	4.8±1.2	0.11
Fasting insulin (µU/mL)	12.3±5.5	21.1±4.4	0.11
Total Cholesterol (mg/dL)	187.9±28.5	191.5±30.3	0.80
Triglycerides (mg/dL)	96.7±31.1	135.1±37.3	0.23
HDL (mg/dL)	57.1±11.9	46.5±10.8	0.09
hs-CRP (mg/L)	9.36±6.5	6.58±4.1	0.32
Diastolic blood pressure (mmHg)	86.1±11.5	83.6±11.5	0.56
Systolic blood pressure (mmHg)	136.7±20.5	131.8±9.9	0.19
MDA (µg/L)	27.6±4.9	54.3±30.2	0.08
TAC (µM/L)	151.8±31.7	126.2±36.3	0.43
mtDNA copy (n)	3553.81±780.1	1185.4±324.2	0.06
Telomeres Length (kbp)	3.47±0.8	1.02±0.4	**0.008**

Abbreviations: BMI (Body Mass Index); HbA1c (Glycosylated Hemoglobin, Type A1C); HOMA (homeostatic model assessment); hs-CRP (high-sensitivity C-reactive protein); MDA (Malondialdehyde); TAC (total antioxidant capacity); mtDNA (mitochondrial DNA). Data are expressed as mean ±Standard Deviation (SD); a p-value<0.05 was considered statistically significant (indicated in bold).
